# The relationship between climate change and the endangered rainforest shrub Triunia robusta (Proteaceae) endemic to southeast Queensland, Australia

**DOI:** 10.1038/srep46399

**Published:** 2017-04-19

**Authors:** Yoko Shimizu-Kimura, Arnon Accad, Alison Shapcott

**Affiliations:** 1 Genecology Research Centre, School of Science, Education and Engineering, Faculty of Science, Health, Education and Engineering, University of the Sunshine Coast, Maroochydore DC, Queensland, 4558 Australia; 2 Queensland Herbarium, Mount Coot-tha Road, Toowong, Queensland, 4066 Australia

## Abstract

Threatened species in rainforests may be vulnerable to climate change, because of their potentially narrow thermal tolerances, small population sizes and restricted distributions. This study modelled climate induced changes on the habitat distribution of the endangered rainforest plant Triunia robusta, endemic to southeast Queensland, Australia. Species distribution models were developed for eastern Australia at 250 m grids and southeast Queensland at 25 m grids using ground-truthed presence records and environmental predictor data. The species’ habitat distribution under the current climate was modelled, and the future potential habitat distributions were projected for the epochs 2030, 2050 and 2070. The eastern Australia model identified several spatially disjunct, broad habitat areas of coastal eastern Australia consistent with the current distribution of rainforests, and projected a southward and upslope contraction driven mainly by average temperatures exceeding current range limits. The southeast Queensland models suggest a dramatic upslope contraction toward locations where the majority of known populations are found. Populations located in the Sunshine Coast hinterland, consistent with past rainforest refugia, are likely to persist long-term. Upgrading the level of protection for less formal nature reserves containing viable populations is a high priority to better protect refugial *T. robusta* populations with respect to climate change.

Climate change is considered a threat to biodiversity as the geographic distribution of suitable habitat for many plant species is expected to change, with range expansions and contractions in some cases increasing the risk of population isolation^1^. Threatened species in rainforest communities are considered to be particularly vulnerable to climatic changes because of traits including narrow thermal tolerances, relatively small population sizes, and/or restricted distributions^2^. Historical climate change in the late Quaternary Period led to significant contraction of Australian rainforest^3^ to refugia which persisted under historical climate fluctuations^4^. However, rapid increases in temperature, and fluctuation in precipitation under projected climate change scenarios have the potential to further contract or expand the habitat range of already patchy rainforest communities in Australia^5^. Subtropical rainforests occupy the interface between tropical and temperate influences; thus projected changes in climatic conditions may impact negatively on these communities^5^. Subtropical rainforest communities in southeast Queensland have a high species diversity and contain many endangered and vulnerable species^6^. However, vegetation in the region is highly fragmented and approximately 56% of the original vegetation has been removed due to extensive land clearing, and many species are now at risk of extinction^7^.

A growing number of studies have modelled the effects of climate change on a large number of plant species around the world^8^,^9^. Species distribution models (SDMs) are commonly used to project future habitat distributions of a species using climate change scenarios^10^,^11^, and can be successfully applied with small numbers of occurrence records^12^. The genus *Triunia* consists of four species, which occur in tropical to subtropical Australian rainforests between north Queensland and northern New South Wales^13^. *Triunia robusta* is a long-lived, subtropical rainforest shrub reported to have less than 750 adult plants remaining across 17 small populations in southeast Queensland^14^, and is ‘Endangered’ as defined by The *Environment Protection and Biodiversity Conservation Act 1999* (EPBC Act), and IUCN Red List of threatened plants^15^. Empirical evidence suggests that *T. robusta* had historically low levels of gene flow and limited dispersal between populations^14^,^16^. *Triunia robusta* may have been more abundant in the landscape prior to habitat fragmentation following European settlement^17^. The potential impact of climate change on the species’ habitat needs investigation to enable planning to support its conservation and persistence in to the future.

This study investigated potential change in the distribution of climatically suitable habitat for *T. robusta* (Proteaceae) under various climate change scenarios, to identify refugia or potential translocation sites for future population management. We specifically investigated: (1) Where are populations of *T. robusta* in eastern Australia currently located, what kind of vegetation communities do they occur in, and how climatically suitable will this habitat be in the future? (2) Is the species distribution range, based on current locations defining the climatic suitability of habitat for *T. robusta*, likely to recede or expand in response to climate change? (3) Which populations within the species distribution range are more likely to persist under projected climate change?

## Methods

### Study area and species occurrence data

**Figure 1:**
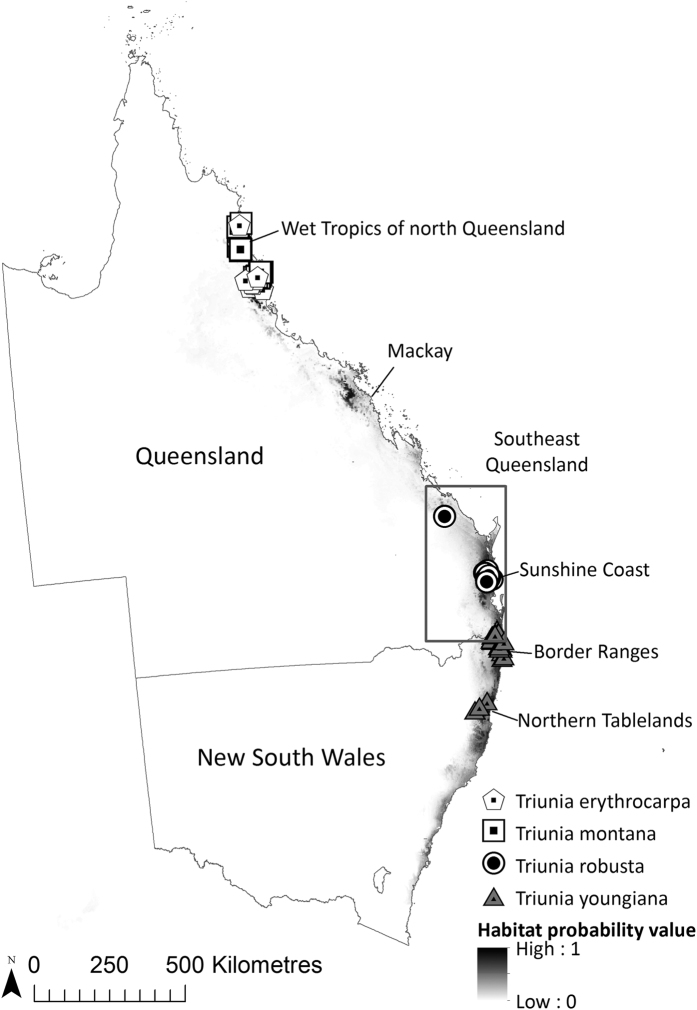
Two nested study areas are shown: (1) eastern Australia (the political jurisdictions of Queensland and New South Wales), and (2) southeast Queensland, generally depicted by a box. The currently known locations of genus *Triunia (T. erythrocarpa, T. montana, T. robusta* and *T. youngiana*) are shown. The eastern Australia bioclimatic model (SDM1) prediction of potential habitat distribution of *T. robusta* under baseline climatic conditions (1990) is also shown. The map was generated in ArcGIS v10.2^24^.

Two nested study areas were investigated: (1) eastern Australia (the political jurisdictions of Queensland and New South Wales), incorporating the currently known distribution of the genus *Triunia* in Australia based on herbarium voucher records^18^, and (2) southeast Queensland, encompassing the known distribution range of *T. robusta*^14^ (boundaries shown in Fig. 1). All 17 known populations were visited, and the location of the approximate centroid of each population was accurately recorded using a handheld GPS device (±10 metres). The majority of the populations cover an area of less than 0.15 ha^14^. Based on Van Proosdij *et al*.^12^, the sample size was within the range of specimen records to develop accurate SDMs^12^. These records were used as the species presence response data (Fig. 1).

### Selection of model predictor variables

We explored 14 climatic and five topographical, geological and geomorphological variables as candidate predictors for the *T. robusta* model that are relevant to plant species (Table 1). The climatic variables included annual (mean annual temperature and annual precipitation), seasonal (quarterly mean temperature and precipitation), and extreme or limiting environmental factors such as temperature of the coldest and warmest month. These variables relate to the biological and eco-physiological tolerance range of plant species^19^. The 14 climate variables were derived from SimCLIM v2.5^20^. SimCLIM is a computer-based climate simulation database system which generates temperature and precipitation anomalies from up to 23 global circulation model (GCM) with spatial resolutions ranging from 100 to 400 km horizontal grids downscaled to a 1 km × 1 km grid resolution for eastern Australia^21^. Future climate surfaces can be generated for six emission scenarios based on the IPCC 4^th^ assessment report (AR4) at yearly intervals up to 2100, using a 1990 baseline climate generated from the interpolation of long-term average monthly weather data^21^,^22^.

Topographic, geological and geomorphological variables are known to be critical factors defining plant distributions because of their influence on soil and substrate conditions supporting plant growth, and therefore have a large impact on persistence^11^. Elevation, slope and aspect (in degrees) were explored as candidate variables in this study, and were generated from the 25 m southeast Queensland Digital Elevation Model (DEM)^23^ in ArcGIS v10.2^24^. In addition, a detailed solid geology of Queensland dataset^25^, and a land zone of Queensland dataset^26^ were obtained. The land zone dataset derives from the Regional Ecosystems (RE) vegetation dataset of Queensland v8.0^27^, which enabled the use of RE envelopes without floristic and community structure. These datasets are of 1:50,000 to 1:100,000 scale, and therefore are detailed enough to segregate into land zones for the purpose of this study.

### Modelling the species distribution with Maxent

We chose Maxent v3.3.3 k^28^,^29^,^30^ to model the habitat distribution of *T. robusta*. Maxent is a software program for modelling species distributions which statistically correlates presence-only records to predictor variables based on the principle of the maximum entropy algorithm^30^. The software has been widely applied for modelling habitat distributions of species under both current and future environments^31^. We developed three SDMs; (1) bioclimatic model of eastern Australia at 250 × 250 m grid resolution using 9-second resolution^32^ DEM (SDM1), (2) bioclimatic model of southeast Queensland at a fine scale 25 × 25 m grid resolution using 25 m southeast Queensland DEM^23^ (SDM2), and (3) the southeast Queensland model which tested bioclimatic, topographical, geological and geomorphological variables at 25 × 25 m grid resolution^23^ (SDM3).

To identify the most informative contributing subsets of variables for each *T. robusta* model, all variables were tested for multicollinearity by conducting Spearman’s rank correlation analysis in SPSS v21.0^33^, with a threshold of *R* > 0.83 to indicate a significant relationship. For significantly correlated pairs of variables, those that made the least contribution to model performance and prediction were omitted based on the Maxent jack knife test results. The results of cross-correlation analysis were reflected accordingly in the final models. The final baseline models (fitted to 1990 climate variables) were then run with five-fold cross validation. Ten thousand background points were automatically and randomly selected by the model; from across eastern Australia for SDM1, and from southeast Queensland for SDM2 and SDM3. Maxent was run with 500 iterations and the logistic output format, which represents the habitat probability values of the target species within the range of 0 to 1 for each grid cell in the model^30^.

### Future climate projections

Six future climate projections were derived using SimCLIM v2.5^20^ for the three futures (2030, 2050 and 2070) and two emissions scenarios (IPCC AR4 A1FI and A2) using an ensemble of five GCMs (MIROC-M, CSIRO-MK3.5, MIUB-ECHO-G, IPSL, and MRI)^22^. These GCMs have proven to be some of the most reliable and commonly used climate models over the Australian region^34^. The A1FI emission scenario is characterised by the highest severity projection of fossil fuel energy sources, rapid economic growth, and the global population growth peaking in the mid-century then declining thereafter^35^. This emission scenario is approximately equivalent to the RCP 8.5, a class of representative concentration pathways (RCP) developed for the IPCC 5^th^ assessment report (AR5)^36^. Conversely, the A2 emission scenario represents high primary energy consumption, with moderate economic growth and continuous population growth^35^. These scenarios were chosen to explore differences between globally oriented and more regionally oriented climate change projections, although the A2 scenario also tracks well above RCP 6.0 and below RCP 8.5^37^. For SDM3, assumptions were made that geology and landform would remain unaltered for the modelling period. The same non-climatic datasets were used for all projections.

Model performance was evaluated by the area under the curve (AUC) in receiver operating characteristic analysis (ROC) of the cross validated model output. An AUC score of 1.0 indicates a statistically valid, perfectly fitting model, while an AUC value of <0.5 indicates a model performing poorly and no better than random^28^. In addition, the true skill statistic (TSS) scores were used for model validation in this study. The TSS takes into account the overall accuracy of the model’s prediction compared to that expected by random chance^38^. Scores range from −1 to +1; values greater than 0.6 indicate good prediction, and values less than 0.2 indicate no better than random^39^.

### Model evaluation and analysis

The baseline model outputs (1990 climates) were used to determine which variables were the best predictors of the species’ range distribution. For each model, distribution maps indicating habitat probability were generated for the three futures (2030, 2050 and 2070) and two emissions scenario (A2 and A1FI). Habitat distribution maps of SDM2 and SDM3 were reclassified to discriminate high and low probability habitat; the minimum probability threshold value was applied based on the mean 10^th^ percentile training presence value across cross-validated models, which uses the 10^th^ percentile of the probability threshold range of the species’ presence records. This non-fixed specific threshold value has been widely used to determine minimum habitat probability threshold values for generating binary maps in many SDM studies^40^.

To develop conservation management recommendations for *T. robusta*, the baseline model outputs (1990 climates) of SDM2 and SDM3, and the regional ecosystems (RE) vegetation dataset of Queensland v8.0^27^ were overlain in order to (1) determine in which rainforest community types *T. robusta* currently occur, and (2) assess the extant area of vegetation in each community type within the projected high probability habitat areas in southeast Queensland, using ArcGIS v10.2^24^.

## Results

### Model evaluation and relative contributions of predictor variables

Five predictor variables were selected to develop the eastern Australia model (SDM1), and four predictor variables were selected for the southeast Queensland bioclimatic/substrate models (SDM2 & SDM3; Table 1). The results of the AUC and TSS analyses indicated that, on average, the models were significantly better than random and have good discrimination ability (SDM1, AUC = 0.996, TSS = 0.861; SDM2, AUC = 0.984, TSS = 0.807; SDM3, AUC = 0.986, TSS = 0.765). The precipitation of the coldest quarter made the highest relative contribution in SDM1 (43.3%), followed by the precipitation of the warmest quarter (25.2%; Table 1). In SDM2, the precipitation of the warmest quarter contributed the most (82.8%), followed by the precipitation of the driest month (16.2%). These two bioclimatic variables were again powerful predictors in SDM3 (57.4%), however as expected, the geological and geomorphological variables also made a strong contribution (42.6%). Rock type such as granitoid, poorly consolidated sediments, and sedimentary rock^25^, and land zones associated with alluvium, fine grated sedimentary rocks, and hills and lowlands on metamorphic/granitic rocks^26^ were selected as generally equating with suitable habitat for *T. robusta* by the model.

### Species habitat distribution under current and future climate conditions

**Figure 2:**
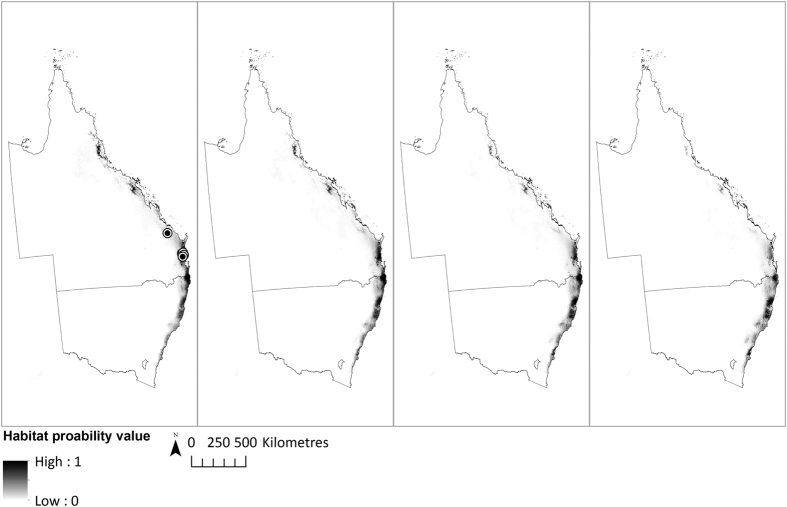
The eastern Australia bioclimatic model (SDM1) projection of potential future habitat distribution of *T. robusta* under IPCC A2 emission scenario. From left to right: (**a**) under baseline climate (1990), (**b**) 2030, (**c**) 2050 and (**d**) 2070. The currently known locations of *T. robusta* sites are shown. All maps were generated in ArcGIS v10.2^24^.

**Figure 3:**
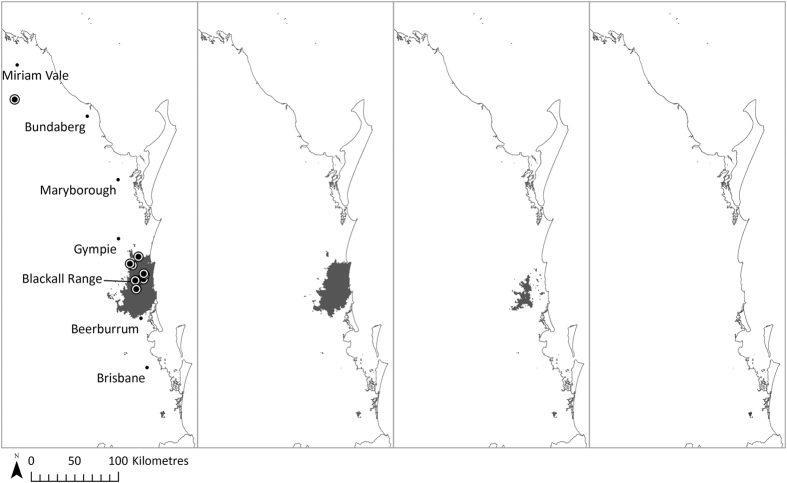
The southeast Queensland bioclimatic model (SDM2) projection of potential high probability future habitat distribution of *T. robusta* under IPCC A2 emission scenario (minimum threshold = 0.2). From left to right: (**a**) under baseline climate (1990), (**b**) 2030, (**c**) 2050 and (**d**) 2070. The currently known locations of *T. robusta* sites are also shown. All maps were generated in ArcGIS v10.2^24^.

**Figure 4:**
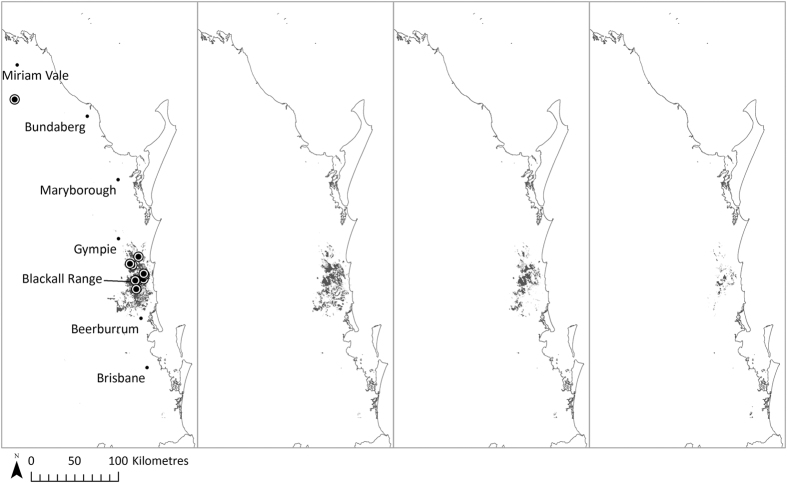
The southeast Queensland bioclimatic and substrate model (SDM3) projection of potential high probability future habitat distribution of *T. robusta* under IPCC A2 emission scenario (minimum threshold = 0.15). From left to right: (**a**) under baseline climate (1990), (**b**) 2030, (**c**) 2050 and (**d**) 2070. The currently known locations of *T. robusta* sites are also shown. All maps were generated in ArcGIS v10.2^24^.

The eastern Australia bioclimatic model (SDM1) identified several major habitat areas for *T. robusta* along coastal eastern Australia under baseline climatic conditions (Fig. 2). The model suggests considerable southward and upslope contraction of the species habitat range particularly in north Queensland under projected climate change, and this was greater with the A1FI high emission scenario. Some novel regions of coastal New South Wales may become suitable habitat by 2070 under either emission scenario. The southeast Queensland bioclimatic model (SDM2) projected high probability habitat for *T. robusta* to be currently found within the greater Sunshine Coast region, covering approximately 2300 km^2^ (Fig. 3 and Table 2). Small habitat patches are also projected in the southern end of the study area. With either emission scenario, a dramatic southward and upslope contraction is projected toward locations where the majority of known populations are currently found. The southeast Queensland substrate model (SDM3) identified much finer, patchy habitat areas within the bioclimatic envelope identified by SDM2, covering approximately 1000 km^2^ (Fig. 4 and Table 2). Although SDM3 followed the same contraction pattern as SDM2, reduction in the extent of suitable habitat in southeast Queensland was projected to be much slower, possibly due to the inclusion of geological and geomorphological variables as proxies for soil and landform potentially moderating local temperature and moisture conditions. As expected, contraction of the suitable habitat areas toward higher, cooler elevations are projected, due to rising temperatures and altered patterns of rainfall consistent with climate change projections in coastal eastern Australia^5^.

### Species occurrence and vegetation community types

Currently *T. robusta* populations occur in six rainforest community regional ecosystem (RE) types, one of which is ‘endangered’ and four of which are ‘of concern’ as defined by the *Vegetation Management Act 1999* (VMA; Table 2). The six RE types are classified structurally as either notophyll vine forests or tall open forests with vine forest understory^41^. The majority of *T. robusta* populations are found in simple notophyll vine forests^41^, usually with abundant Bangalow palm, *Archontophoenix cunninghamiana* (H.Wendl.)^42^, on Mesozoic to Proterozoic igneous rocks. The southeast Queensland bioclimatic model (SDM2) found that, under baseline climatic conditions, approximately 59% (1300 km^2^) of the high probability habitat areas are associated with areas that have been cleared. Only 8% (200 km^2^) of the high probability habitat is found within the six RE types which currently accommodate *T. robusta*. The remaining 33% (800 km^2^) are associated with RE types in which the species has never been reported, although these do include notophyll vine forests and other endangered ecosystems (Table 2). The contribution of high probability habitat associated with the six RE types was further reduced when geological and geomorphological variables were incorporated in the model (SDM3), covering only 12.0% (100 km^2^) of the high probability habitat areas within the southeast Queensland region under baseline climatic conditions.

## Discussion

Whilst modelling the species habitat distribution in response to climate change is an active field of research, SDMs are subject to uncertainties and limitations. For instance, accuracy of the species location data is known to influence model projection outcomes^43^. In this study, the species location data precision level was high. However, obtaining additional presence locations from the full extent of each population area could have improved the model validity, although using natural occurrence data in SDMs might under-predict potential distribution range, as plant species do not necessarily fully encompass their fundamental niche^44^. Variable selection and accuracy of datasets are also a potential source of uncertainty in SDMs^45^. In this study, only climatic variables were explored in SDM1 and SDM2 to model the broad climatic domain. However, exclusion of other environmental variables may have resulted in models predicted to increase the likelihood of climate impacts. In SDM3, geological and geomorphological variables were coupled with climatic variables, and the model predicted much slower climate impacts. This demonstrates that local conditions could potentially buffer (decouple) regional climate^46^, although coupling fine-resolution substrates (25 × 25 m grid) and low-resolution climate (1 × 1 km grid) might result in models weighted toward substrate variables. Moreover, soil and vegetation dataset in future years that are consistent with IPCC scenarios were not available at the time of the study. The predictive power of the models could be further improved by inclusion of better representations of substrates in future studies. The climate projections are subject to uncertainty as different combinations of downscaling methods, GCMs and emission scenarios may yield different outcomes^47^. In this study, an ensemble of five GCMs was used, and therefore these uncertainties were averaged at the time of the climate projection modelling. It has been highlighted that the majority of SDM studies seldom take into account species demographic factors^48^. The SDMs developed in this study did not take into account adaptive potential of *T. robusta*, as given the study time frame (1990–2070) and the species generation time (44 years)^14^, there would not be sufficient time for genetic adaptation^49^. Despite these limitations, the results of this study demonstrated that SDMs were useful to evaluate general trends in the mainly climatic habitat distribution of *T. robusta* at both continental and regional scales under climate change.

Many rainforest Proteaceae in Australia have restricted distributions and are confined to rainforest remnants^50^. This study identified several spatially disjunct broad habitat areas of potential climatic suitability for the endangered shrub *T. robusta* (Proteaceae) under baseline climatic conditions, including the Wet Tropics of north Queensland, northwest of Mackay in central Queensland, the greater Sunshine Coast region in southeast Queensland, the Border Ranges on the state border of Queensland and New South Wales, and the Northern Tablelands in northern New South Wales located along costal eastern Australia. These areas are consistent with the current distribution of the major tropical and subtropical rainforest remnants of eastern Australia^51^. Interestingly, the potential habitat areas identified where *T. robusta* has not been recorded are currently occupied by its congeners *T. youngiana, T. erythrocarpa* and *T. montana*, suggesting that species in the genus *Triunia* may share a similar climatic niche, although they may differ in substrate fidelity. Hence, the eastern Australian model developed in this study may be a better predictor for the genus *Triunia* than the individual species within the genus, suggesting the need to test a hypothesis of speciation in the genus following geographical isolation.

*Triunia robusta* is a narrow endemic, being confined to fragmented subtropical rainforest remnants in southeast Queensland^14^. Fifteen out of 17 currently known populations occur in RE types that are either ‘endangered’ or ‘of concern’ as defined by the *Vegetation Management Act 1999* (VMA). Within the species distribution range, the highest concentration of high probability habitat areas under baseline climatic conditions is found between Gympie and Beerburrum within the greater Sunshine Coast, where the majority of the currently known populations occur. These habitat areas are concentrated in the southern part of the species distribution range, which highlights the potential vulnerability of northern range populations to climate change. The extent of high probability habitat predicted to be associated with REs known to accommodate *T. robusta* was low overall, and is set amongst a landscape matrix comprising up to 59% of habitat that has been cleared or degraded. This suggests that landscape scale interruptions to ecological processes are likely to impact on the persistence of this species under climate change.

Several studies argue that contemporary climate change over the last 100 years is already causing changes in the habitat distribution of many species^52^. Habitat distributions of many plant species in the tropical and subtropical region of coastal eastern Australia are projected to show southward range expansion and upslope contraction due to rising temperatures and altered patterns of rainfall^5^. Dramatic climate-driven southward expansion of the potential habitat range was projected along the coastal eastern Australia for *T. robusta*, with the southern border of the species habitat range projected to extend to southern New South Wales. Suitable habitat areas in north Queensland are projected to be the first and worst affected, with habitat ranges showing substantial southward and upslope contraction, which may impact on the survivorship, vulnerability to disease, and reproductive success of existing populations of its congeneric species, given their limited dispersal ability to track suitable habitat. The results also indicate a possible emergence of novel climatically suitable habitat areas in central New South Wales, which may become viable translocation sites in the future, if substrate conditions are also found to be suitable.

A recent study by Weber *et al*.^53^ identified five centres of rainforest plant endemism in southeast Queensland and northern New South Wales, including the Sunshine Coast hinterland, which may have functioned as refugia for subtropical rainforest species in the past^53^. Powell *et al*.^54^ also suggest that the co-occurring, related subtropical rainforest endemic tree genus *Macadamia* (Proteaceae) may have persisted in refugial rainforest in southeast Queensland under the historical climatic fluctuations^54^. This study of *T. robusta* found that habitat areas within the species’ known distribution range are projected to show substantial upslope contraction toward areas consistent with past refugia, with the Blackall Range in the Sunshine Coast hinterland likely to be the species’ core habitat for the next 55 years. This is consistent with the area where the majority of the currently known, large and relatively stable populations occur^14^. Endemic species which currently occupy areas considered to be past refugia may be less likely to be affected greatly by future climate change at least in the short term^54^.

Plant species that have lower reproductive rates and relatively long generation time such as *T. robusta* are thought to have longer lag times in response to climate change, and so be potentially isolated in unsuitable habitat as a future consequence^55^. The results of this study suggest that populations that are located in the Blackall Range on the Sunshine Coast hinterland, consistent with past rainforest refugia are likely to persist under future climate change. These include 10 currently known populations; three of which are located in Triunia National Park, six located in Mapleton State Forest, and one located in Wappa Dam Water Reserve^14^. The Rocky Creek division of Mapleton State Forest Reserve and Wappa Dam falls are of particular importance, as these areas contain the three largest, most viable populations of *T. robusta* (>300 individual plants)^14^. However, these less formal reserves currently receive a low level of long-term protection compared to areas protected by legislation and included in Australia’s National Reserve System^56^. Upgrading the level of protection for the portion of the State Forest Reserve and Water Reserve containing *T. robusta* populations in the Blackall Range area will be a high priority to better protect the species over the long-term, particularly with respect to climate change.

This study utilised SDM as a tool to assess general trends of climate change impacts on the current and future habitat distribution of *T. robusta* at both continental and regional scales, and to suggest potential conservation priority areas for the long term persistence of the species. Further refinement of the SDMs with integration of higher resolution topographically-downscaled climate data^57^ and better representations of substrate variables^58^, as well as species ecological dynamics^59^,^60^ is necessary for better discrimination of future suitable habitat areas of *T. robusta* for predicting species long-term persistence under climate change, and confirm management recommendations.

## Additional Information

**How to cite this article**: Shimizu-Kimura, Y. *et al*. The relationship between climate change and the endangered rainforest shrub *Triunia robusta* (Proteaceae) endemic to southeast Queensland, Australia. *Sci. Rep.* **7**, 46399; doi: 10.1038/srep46399 (2017).

**Publisher's note:** Springer Nature remains neutral with regard to jurisdictional claims in published maps and institutional affiliations.

## Figures and Tables

**Table 1:** A list of predictor variables explored for the *T. robusta* models and their percentage contribution to the final models.

Predictor variable	Unit	SDM1			SDM2			SDM3		
		Min	Max	%	Min	Max	%	Min	Max	%
Annual mean temperature	C˚	—	—	—	—	—	—	—	—	—
Max temperature of warmest month	C˚	27	29	9.7%	27	29	0.2%	—	—	—
Min temperature of coldest month	C˚	7	9	9.6%	7	9	0.8%	—	—	—
Mean temperature of wettest quarter	C˚	—	—	—	—	—	—	—	—	—
Mean temperature of driest quarter	C˚	—	—	—	—	—	—	—	—	—
Mean temperature of warmest quarter	C˚	—	—	—	—	—	—	—	—	—
Mean temperature of coldest quarter	C˚	13.3	15.3	12.3%	—	—	—	—	—	—
Annual precipitation	mm	—	—	—	—	—	—	—	—	—
Precipitation of wettest month	mm	—	—	—	—	—	—	—	—	—
Precipitation of driest month	mm	—	—	—	36	46	16.2%	36	46	14.6%
Precipitation of wettest quarter	mm	—	—	—	—	—	—	—	—	—
Precipitation of driest quarter	mm	—	—	—	—	—	—	—	—	—
Precipitation of warmest quarter	mm	489	720	25.2%	489	720	82.8%	489	720	42.8%
Precipitation of coldest quarter	mm	147	267	43.3%	—	—	—	—	—	—
Elevation	m	—	—	—	—	—	—	—	—	—
Slope	degree	—	—	—	—	—	—	—	—	—
Aspect	degree	—	—	—	—	—	—	—	—	—
Geology (SDM3 only)	Class	Granitoid, poorly consolidated sediments, and sedimentary rock (29.5%)								
Land zone (SDM3 only)	Class	Alluvium, hills and lowlands on metamorphic/granitic rocks (13.1%)								

The minimum and maximum values and selected classification of each predictor variable to the final models are given.

**Table 2:** Six vegetation community types identified to accommodate *T. robusta* based on the regional ecosystems (RE) vegetation dataset of Queensland v 8.0^27^ and SDM2/SDM3 output.

RE type	Description	Sites	SDM2	SDM3
			km^2^ (%Area)	km^2^ (%Area)
12.11.10	Notophyll vine forest with *Araucaria cunninghamii* on metamorphics - interbedded volcanics (of concern)	2	19.1 (0.9)	0.1 ( < 0.1)
12.12.1	Simple notophyll vine forest (gully vine forest) usually with abundant *Archontophoenix cunninghamiana* on Mesozoic to Proterozoic igneous rocks (of concern).	10	45.1 (2.0)	47.7 (4.8)
12.12.16	Notophyll vine forest on Mesozoic to Proterozoic igneous rocks (no concern)	2	22.1 (1.0)	22.8 (2.3)
12.3.1	*Eucalyptus grandis* tall open forest with rainforest dominated but mixed species understorey on alluvial plains (endangered)	1	21.6 (0.9)	13.0 (1.3)
12.3.2	Gallery rainforest (notophyll vine forest) on alluvial plains (of concern)	1	42.7 (1.9)	17.5 (1.8)
12.9–10.1	Tall open forest often with *Eucalyptus resinifera, E. grandis, E. robusta, Corymbia intermedia* on sedimentary rocks (of concern)	1	28.7 (1.3)	17.6 (1.8)
Other	Other RE types (including rainforests)	0	751.3 (33.4)	546.4 (54.9)
Non-remnant	Cleared, plantations, etc	0	1315.7 (58.6)	329.8 (33.1)
Total Area		17	2246.3 (100)	994.9 (100)

Area abundance of each RE vegetation type within the species’ high probability habitat is given in km^2^ and percentage in parentheses. Area abundance of vegetation communities other than the seven RE types within the species’ high probability habitat is also given (other RE types and non-remnant).
